# Solitary lung adenocarcinoma: follow-up CT, pathological-molecular characteristics, and surgical prognosis for different morphological classifications

**DOI:** 10.1186/s13244-023-01563-x

**Published:** 2023-11-27

**Authors:** Hong-fan Liao, Xing-tao Huang, Xian Li, Fa-jin Lv, Tian-you Luo, Qi Li

**Affiliations:** 1 Department of Radiology, the First Affiliated Hospital of Chongqing Medical University, No. 1 Youyi Road, Yuzhong District, Chongqing, 400016 China; 2 College of Medical Informatics, Chongqing Medical University, Chongqing, 400016 China; 3 Department of Radiology, the Fifth People’s Hospital of Chongqing, Chongqing, 400062 China; 4 Department of Pathology, Chongqing Medical University, Chongqing, China

**Keywords:** Lung adenocarcinoma, Computed tomography, Gene mutation, Histological subtype, Prognosis

## Abstract

**Objective:**

To investigate the dynamic changes during follow-up computed tomography (CT), histological subtypes, gene mutation status, and surgical prognosis for different morphological presentations of solitary lung adenocarcinomas (SLADC).

**Materials and methods:**

This retrospective study compared dynamic tumor changes and volume doubling time (VDT) in 228 patients with SLADC (morphological types I–IV) who had intermittent growth during follow-ups. The correlation between the morphological classification and histological subtypes, gene mutation status, and surgical prognosis was evaluated.

**Results:**

Among the 228 patients, 66 (28.9%) were classified as type I, 123 (53.9%) as type II, 16 (7%) as type III, and 23 (10.1%) as type IV. Type I had the shortest VDT (254 days), followed by types IV (381 days) and III (501 days), and then type II (993 days) (*p* < 0.05 each). Type I had a greater proportion of solid/micropapillary-predominant pattern than type II, and the lepidic-predominant pattern was more common in type II and III than in type I (*p* < 0.05 each). Furthermore, type II and IV SLADCs were correlated with positive epidermal growth factor receptor mutation (*p* < 0.05 each). Lastly, the Kaplan–Meier curves showed that the disease-free survival was longest for patients with type II tumors, followed by those with type III and IV tumors, and then those with type I tumors (*p* < 0.001 each).

**Conclusion:**

A good understanding of the natural progression and pathological-molecular characteristics of different morphological SLADC types can help make accurate diagnoses, develop individual treatment strategies, and predict patient outcomes.

**Critical relevance statement:**

A good understanding of the natural progression and pathological-molecular characteristics of different morphological solitary lung adenocarcinoma types can help make accurate diagnoses, develop individual treatment strategies, and predict patient outcomes.

**Key points:**

• Type I–IV solitary lung adenocarcinomas exhibit varying natural progression on serial CT scans.

• Morphological classification of solitary lung adenocarcinomas predicts histological subtype, gene status, and surgical prognosis.

• This classification of solitary lung adenocarcinomas may help improve diagnostic, therapeutic, and prognosticating abilities.

**Graphical Abstract:**

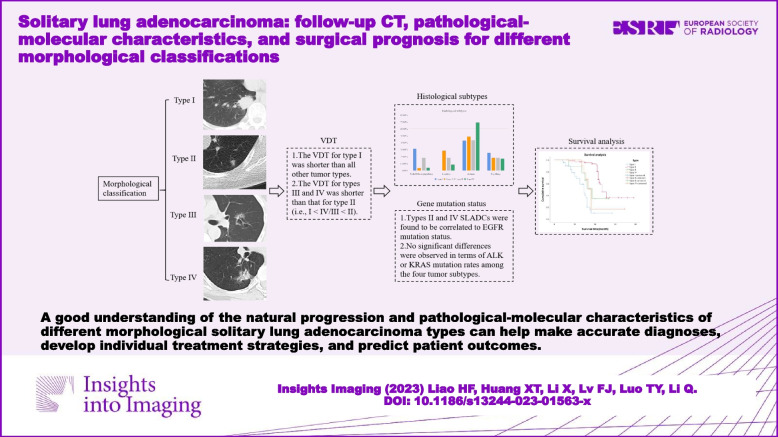

**Supplementary Information:**

The online version contains supplementary material available at 10.1186/s13244-023-01563-x.

## Introduction

Lung cancer is the leading cause of cancer-related mortality globally, with lung adenocarcinoma (LADC) being the commonest histological subtype [1]. Although computed tomography (CT) is the first-line imaging tool for the screening and diagnosis of lung cancer, LADC, which is known for its heterogeneity, can exhibit diverse manifestations on CT, varying from subsolid to solid nodules and masses, and single or multifocal lesions [2, 3]. Previously, we reported four types of solitary LADCs (SLADCs) based on CT imaging—type I (solid nodule or mass), type II (subsolid nodule or mass, including pure ground-glass nodule [GGN] and mixed GGN), type III (cystic airspace), and type IV (focal consolidation involving < 50% of the area of a lobe) [4]. Familiarity with the protean imaging appearances of SLADC is essential to ensure diagnostic accuracy.

It has been observed that the smaller a tumor is, the less typical CT signs it displays, thereby obscuring the correct diagnosis [5]; hence, recognizing the trends of progression and growth for small tumors via CT follow-ups can provide valuable clues for early diagnosis of lung cancer [6–8]. Being the most frequent subtype of LADC, invasive LADC usually displays five growth patterns—lepidic, acinar, papillary, solid, and micropapillary, which often manifest in a combination. Furthermore, it has been pointed out that pattern-based staging of invasive LADC is closely related to patient outcomes [9–11]. Since targeted therapy provides higher success rates and reduced complications and toxicity in patients with advanced lung cancer and driver gene mutations (e.g., epidermal growth factor receptor [EGFR] mutation), identifying the relationship between CT morphological features and driver gene mutations in LADC may be advantageous for therapeutic decision-making in patients where obtaining pathologic specimens is difficult [12–14].

So far, surgical resection remains the primary therapy for early LADC; in these patients, a knowledge of the correlation between imaging features and postoperative survival outcomes may help guide their management. In our clinical experience, SLADCs with different morphological appearances may exhibit different natural courses, pathological and molecular characteristics, and survival prognoses. A good understanding of these features may allow clinicians to choose the optimal treatment strategy and prognostication [15–17]. To our knowledge, the aforementioned characteristics have not been fully elucidated for patients with type I–IV SLADCs. Thus, this study aimed to investigate the dynamic changes during follow-up CT, histological subtypes, gene mutation status, and surgical prognosis for different morphological classifications of SLADCs.

## Materials and methods

### Patients

This retrospective study was approved by the institutional review board of our institution; the need for informed consent was waived by the review board. For this study, we selected patients with SLADC admitted to our institution between January 2015 and July 2022 based on the following inclusion criteria: (1) patients undergoing ≥ 2 follow-up chest CT scans at an interval (namely, between the first and second CT examinations, second and third CT examinations, and so on) of ≥ 90 days before pathological confirmation; (2) tumors with intermittent growth defined when one of the following conditions was observed: (a) a ≥ 2-mm increase in diameter on follow-up CTs compared to initial CT; (b) solid portions in part-solid GGNs increased by ≥ 2 mm compared to initial CT, or (c) new solid portions occurring within the pure GGNs; (3) patients were pathologically confirmed for LADC. Patients were excluded if they had a history of antitumor treatment before chest CT examination, or if the imaging quality was poor due to obvious artifacts.

Baseline data for all patients, including age at the time of diagnosis, sex, smoking history, and diagnostic method used, were obtained from the electronic medical record system. Patients who underwent surgery were selected for the survival analysis, and their follow-up information was obtained from outpatient visits and telephonic follow-ups. Disease-free survival (DFS) was defined as the time from the date of surgery to the date of recurrence of the SLADC or death due to any cause or to the date of the last follow-up without progression. The last follow-up date for the study patients was February 16, 2023.

### Protocol for CT examination

All patients were imaged by a Discovery 750 HD CT (GE Healthcare), a Somatom Perspective (Siemens Healthcare), or a Somatom Definition FLASH (Siemens Healthcare) scanner. The following scanning parameters were used: tube voltage = 110–120 kVp; tube current = 50–250 mAs (automatic tube current modulation technology); rotation time = 0.5 s; pitch = 0.875–1.50; slice thickness = 0.625–1.25 mm; and interval = 0.625–1.25 mm on axial images. The patient was positioned in a supine (lying on their back) posture and underwent a craniocaudal direction scan (from head to toe). The scan was performed at the end of inspiration (breath in) and completed within a single breath-hold. The imaging covered the area from the thoracic inlet (upper chest) to just below the costophrenic angle (where the diaphragm meets the ribs).

### CT image analysis

All CT data were reviewed by two radiologists (having 9 and 15 years of experience in chest CT imaging) on a picture archiving and communication (PACS) workstation (Vue PACS, Carestream) individually; any differences in CT manifestations of tumors were resolved through discussion. First, all tumors were classified into four types (types I–IV) according to their morphological findings on CT; next, the morphological evolution of the four categories was studied during follow-up CTs. The CT features analyzed and measured are displayed in Table 1, and all specific CT features are depicted in Supplementary material 1.

**Table 1:** CT features analyzed and measured for type I–IV SLADCs

	Type I	Type II	Type III	Type IV
Characteristics	Location Size Shape Lobulation Spiculation Air bronchogram sign Air space Pleural retraction Pleural attachment Vessel convergence sign Lymphadenopathy Pleural effusion	Location Size Shape CT value of tumor Lobulation Spiculation Air bronchogram sign Air space Pleural retraction Pleural attachment Vessel convergence sign Lymphadenopathy Pleural effusion	Location Size Shape Cyst walls thickness Septation within the cyst GGO component Pleural retraction Pleural attachment Vessel convergence sign Lymphadenopathy Pleural effusion	Location Size Shape CT value of tumor Air bronchogram sign Air space GGO component Pleural retraction Pleural attachment Vessel convergence sign Lymphadenopathy Pleural effusion

*CT* computed tomography, *GGO* ground-glass opacity, *SLADC* solid lung adenocarcinoma

### Volume measurement and calculating the volume doubling time

One of the radiologists performed three-dimensional manual segmentation of tumors using volume measurement software in the PACS workstation. For each patient, the volume of interest was cautiously drawn along the tumor margins on axial CT images layer by layer, covering the whole tumor contour; accordingly, the volume of the segmented tumor was automatically calculated. To evaluate intra-observer repeatability, the radiologist randomly selected 50 patients and repeated the ROI delineation 1 month later. To evaluate inter-observer repeatability, the second radiologist randomly selected 50 patients and performed the ROI segmentation independently. Thereafter, the intraclass correlation coefficient (ICC) was calculated to evaluate the stability and reproducibility of volume measurement; ICC values > 0.75 indicated good reliability.

The volume doubling time (VDT) of tumors was calculated using the modified Schwartz equation of an exponential growth model as follows:

$$\mathrm{VDT}=\frac{[\mathrm{log}2 \times \mathrm{ T}]}{[\mathrm{log}(\mathrm{V}2/\mathrm{V}1)]}$$

where V1 and V2 represent the tumor volumes in the initial and last CT scans before the antitumor therapy, respectively, and T represents the time interval between these two CT scans [18].

### Histochemical examination

All specimens were stained with hematoxylin and eosin and analyzed by two experienced pathologists together who were blinded to patient data, and decisions on pathological diagnosis were reached through consensus. In accordance with the World Health Organization’s 2015 classification of LADC, the percentage of each growth pattern (acinar, lepidic, papillary, solid, and micropapillary) in the tumor was evaluated semi-quantitatively in 5% increments, and the pattern with the largest percentage was designated as the dominant pattern [19].

### Gene mutation testing

Surgical specimens were analyzed using Amoy Diagnostics’ commercially available kits which are based on the amplified refractory mutation system real-time polymerase chain reaction technology to qualitatively detect and recognize gene mutation or fusion in the SLADC, including EGFR, anaplastic lymphoma kinase (ALK), and the Kirsten rat sarcoma viral oncogene (KRAS).

### Statistical analysis

All analyses were performed using SPSS version 25.0 (IBM Corp.). Categorical data were evaluated using the chi-squared test, and Bonferroni correction was used for pairwise comparison. Continuous data were first tested for normality using the Kolmogorov–Smirnov test, mean ± standard deviation was described for the normally distributed data, and ANOVA testing was used for between-group comparisons. Non-normally distributed data were represented by median (interquartile range, IQR), and the Kruskal–Wallis *H* test was used for between-group analysis. A *p* value of < 0.05 was used to determine statistical significance. The intraclass correlation coefficient (ICC) was used to evaluate the diagnostic consistency of CT features of tumors, and an ICC value > 0.75 was regarded as a good agreement between the two radiologists. Additionally, the Kaplan–Meier survival analysis and log-rank test were performed to compare the DFS of patients with different morphological types who underwent surgical resection. For type III tumors, Spearman rank correlation analysis was performed to evaluate the correlation between the thickness of cystic walls before operation and DFS of patients.

## Results

### Baseline characteristics

**Fig. 1:**
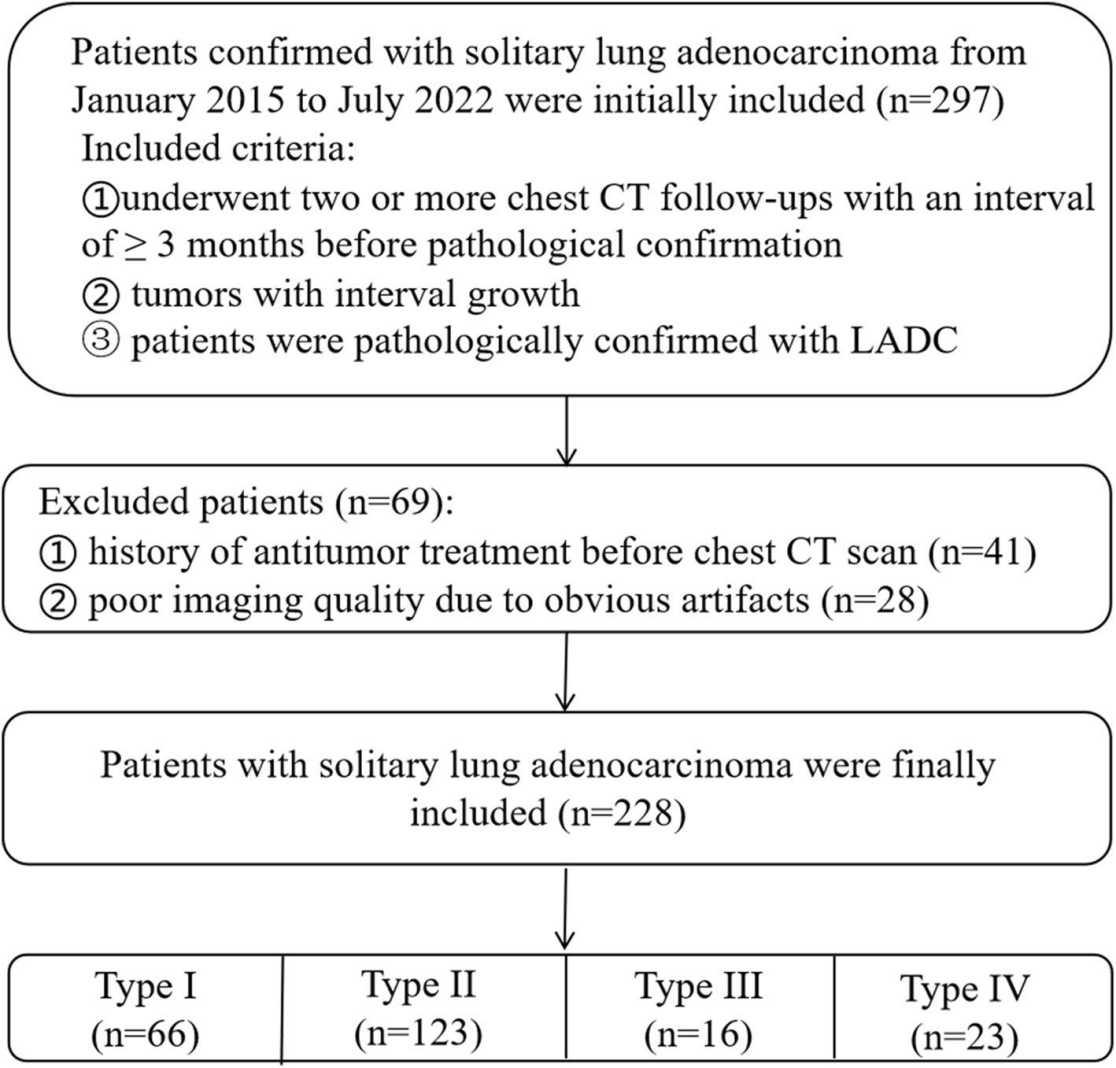
Selection flowchart for this study

A total of 297 patients with SLADC were initially screened for inclusion, of which 41 were excluded because of a history of antitumor therapy before the chest CT scan and 28 owing to poor imaging quality. Finally, 228 patients with SLADC were included for analysis. The patient selection flowchart is shown in Fig. 1. Among the 228 patients, 66 (28.9%) were classified as type I (mean age: 61.64 ± 7.75 [range: 42–80] years; 34 males and 32 females; 45 smokers and 21 nonsmokers), 123 (53.9%) as type II (mean age: 59.27 ± 10.47 [range: 30–81] years; 50 males and 73 females; 39 smokers and 84 nonsmokers), 16 (7%) as type III (mean age: 58.75 ± 6.93 [range: 49–72] years; 11 males and 5 females; 8 smokers and 8 nonsmokers), and 23 (10.1%) as type IV (mean age: 65.21 ± 8.39 [range: 50–83] years; 5 males and 18 females; 10 smokers and 13 nonsmokers) (Table 2). The diagnostic methods included surgical resection, fiberoptic bronchoscopy, puncture biopsy, and cytology, with surgical resection being the most employed method (*n* = 202/228; 88.6%). A total of 134 patients had undergone two follow-up CT scans, 61 had undergone three follow-up CT scans, 21 had undergone four follow-up CT scans, 7 had undergone five follow-up CT scans, 4 had undergone six follow-up CT scans, and 1 patient had undergone eight follow-up CT scans. The mean tumor size was 24.37 ± 9.29 mm in the initial CT scan and 29.43 ± 12.1 mm in the last CT scan.

**Table 2:** Baseline characteristics of patients

Characteristics	All patients (*n* = 228)	Type I (*n* = 66)	Type II (*n* = 123)	Type III (*n* = 16)	Type IV (*n* = 23)
Age (years)					
Mean	60.46 ± 9.50	61.64 ± 7.75	59.27 ± 10.47	58.75 ± 6.93	65.21 ± 8.39 50–
Range	30–83	42–80	30–81	49–72	83
Sex					
Male	100 (43.9%)	34 (51.5%)	50 (40.7%)	11 (68.8%)	5 (21.7%)
Female	128 (56.1%)	32 (48.5%)	73 (59.3%)	5 (31.2%)	18 (78.3%)
Smoking					
Smokers	105 (46.0%)	45 (68.2%)	39 (31.7%)	8 (50.0%)	10 (43.5%)
Nonsmokers	123 (54.0%)	21 (31.8%)	84 (68.3%)	8 (50.0%)	13 (56.5%)
Diagnostic method					
Surgical resection	202 (88.6%)	51 (77.3%)	112 (91.1%)	16 (100%)	23 (100%)
Fibroscopic bronchoscopy	15 (6.6%)	12 (18.2%)	3 (2.4%)	0 (0)	0 (0)
Puncture biopsy	10 (4.4%)	2 (3%)	8 (6.5%)	0 (0)	0 (0)
Cytology	1 (0.4%)	1 (1.5%)	0 (0)	0 (0)	0 (0)
Number of CT scans					
Two	133 (58.3%)	37 (56.1%)	72 (58.5%)	10 (62.5%)	14 (60.9%)
Three	61 (26.8%)	19 (28.8%)	31 (25.2%)	4 (25.0%)	7 (30.4%)
Four	20 (8.7%)	8 (12.1%)	9 (7.3%)	1 (6.25%)	2 (8.7%)
Five	8 (3.5%)	1 (1.5%)	6 (4.9%)	1 (6.25%)	0 (0)
Six	4 (17.5%)	0 (0)	4 (3.3%)	0 (0)	0 (0)
Seven	1 (0.4%)	0 (0)	1 (0.8%)	0 (0)	0 (0)
Eight	1 (0.4%)	1 (1.5%)	0 (0)	0 (0)	0 (0)
Tumor size (mm)					
Initial CT scan	24.37 ± 9.29	15.01 ± 7.46	12.37 ± 5.15	31.68 ± 12.03	38.4 ± 12.5
Last CT scan before therapy	29.43 ± 12.1	19.87 ± 8.2	15.25 ± 6.34	33.4 ± 12.26	49.2 ± 21.6

*CT* computed tomography, *SLADC* solid lung adenocarcinoma

### Observer reproducibility

The agreement between the two radiologists was pretty good for all CT features (Supplementary Table 1). The ICC values for CT features of type I tumors ranged from 0.898 to 1.000; those for CT features of type II tumors ranged from 0.899 to 1.000; those for CT features of type III tumors ranged from 0.828 to 1.000; and those for CT features of type IV tumors ranged from 0.904 to 1.000 (all* p* < 0.001).

### Imaging evolution and VDT of different SLADC types

**Fig. 2:**
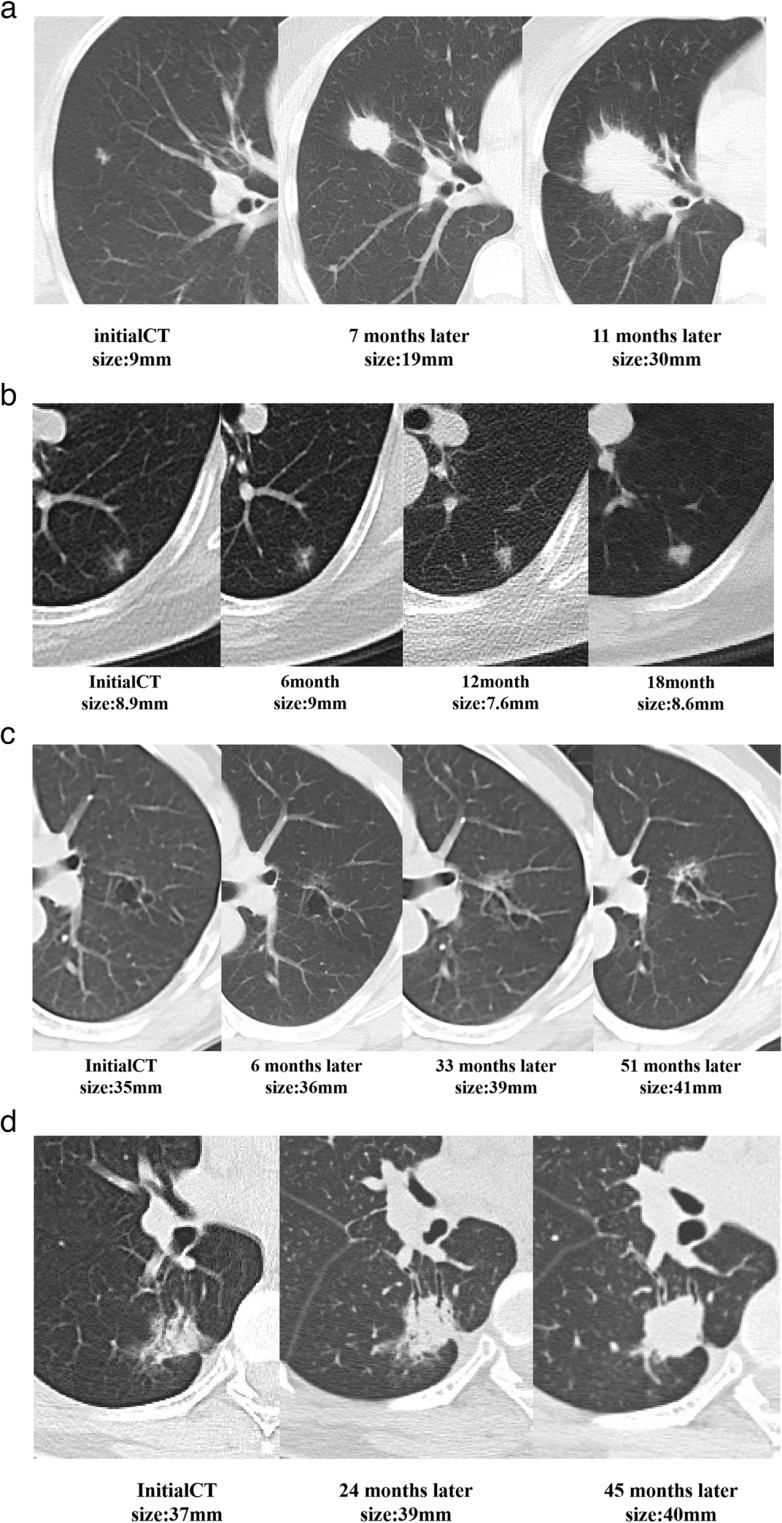
Axial CT images show the evolution of type I-IV SLADCs during follow-ups. **a** Sequential images in a 47-year-old woman who had LADC show that the tumor enlarged over time with lobulation, spiculation, and pleural retraction appeared. **b** Sequential images in a 60-year-old woman who had LADC show that the tumor was stable firstly, then decreased with solid components and density increased, and then increased again. **c** Sequential images in a 65-year-old man who had LADC show that the cystic airspace enlarged, cystic walls thickened, and wall nodules appeared. **d** Sequential images in a 58-year-old woman who had LADC show that a focal consolidation enlarged over time, with attenuation increased as well as air bronchogram and GGO component gradually disappeared, and eventually became a solid mass

In type I tumors, the tumor diameter enlarged over time, i.e., lobulation, spiculation, air bronchogram sign, pleural retraction, vessel convergence sign, lymphadenopathy, and pleural effusion gradually appeared in the tumor (Fig. 2a). Most of the type II tumors (*n* = 96/123; 78.0%) enlarged over time with increased density or solid components, whereas the remaining tumors [*n* = 27/123; 22.0%] were fluctuating between regression and enlargement, with increasing density or solid components (Fig. 2b). Additionally, lobulation, spiculation, and the air bronchogram sign also gradually appeared in these tumors. For type III, the diameter of cystic airspace enlarged over time—cystic walls thickened, and wall nodules appeared; eventually, the cystic airspace narrowed or disappeared and the tumor became a solid mass (observed in two patients) (Fig. 2c). In type IV SLADC, the tumor enlarged over time—the attenuation increased while the air bronchogram sign, air space, and GGO component gradually disappeared, and the lesion ultimately became a solid mass (Fig. 2d).

We observed good inter- and intra-observer reliability for tumor volume measurement (ICCs = 0.880 and 0.921, respectively). The median VDT was 601 (IQR = 975) days (range = 45–2750 days) for all patients. Type I tumors had the shortest median VDT (254 [IQR = 201] days; range = 45–1966 days), followed by type IV (median = 381 [IQR = 590] days; range = 60–2056 days), type III (median = 501 [IQR = 777] days; range = 95–2113 days), and type II (median = 993 [IQR = 812] days; range = 112–2750 days). The VDT for type I was shorter than all other tumor types (*p* < 0.05 each); furthermore, the VDT for types III and IV was shorter than that for type II (i.e., I < IV/III < II) (*p* < 0.05 each). However, no statistically significant difference was observed between the VDT for types III and IV (*p* > 0.05).

### Correlation between histological subtypes and morphological classification of SLADC

**Fig. 3:**
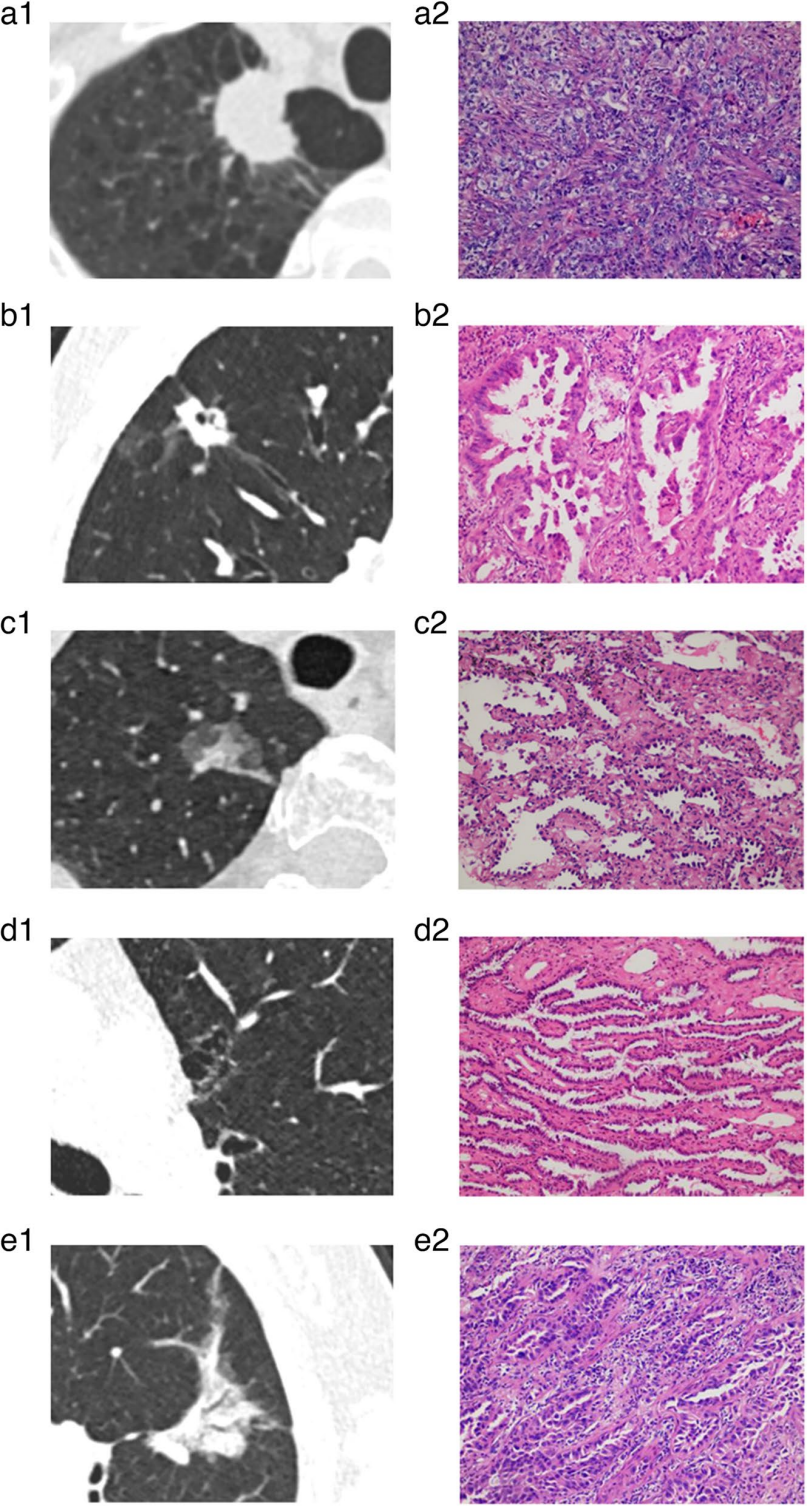
The histological subtypes of SLADC and their correlation with morphological classifications. **a** R-SLADC in a 59-year-old man with type I. **a-1** Axial CT images of lung window indicate a solid mass with evident lobulation and spiculation. **a-2** Photomicrograph (H&E staining, × 200) confirmed SLADC with a solid-predominant growth pattern. **b** R-SLADC in a 63-year-old man with type I. **b-1** Axial CT images of the lung window indicate a solid mass with evident air space and pleural attachment. **b-2** Photomicrograph (H&E staining, × 200) confirmed SLADC with a micropapillary-predominant growth pattern. **c** R-SLADC in a 76-year-old woman with type II. **c-1** Axial CT images of the lung window indicate a subsolid mass with evident pleural attachment. **c-2** Photomicrograph (H&E staining, × 200) confirmed SLADC with a lepidic-predominant growth pattern. **d** L-SLADC in a 55-year-old man with type III. **d-1** Axial CT images of the lung window indicate a cystic airspace attached to the mediastinum. **d-2** Photomicrograph (H&E staining, × 200) confirmed SLADC with a papillary-predominant growth pattern. **e** L-SLADC in a 67-year-old woman with type IV. **e-1** Axial CT images of the lung window indicate a focal consolidation with evident GGO component and air bronchogram sign. **e-2** Photomicrograph (H&E staining, × 200) confirmed SLADC with an acinar-predominant growth pattern

**Fig. 4:**
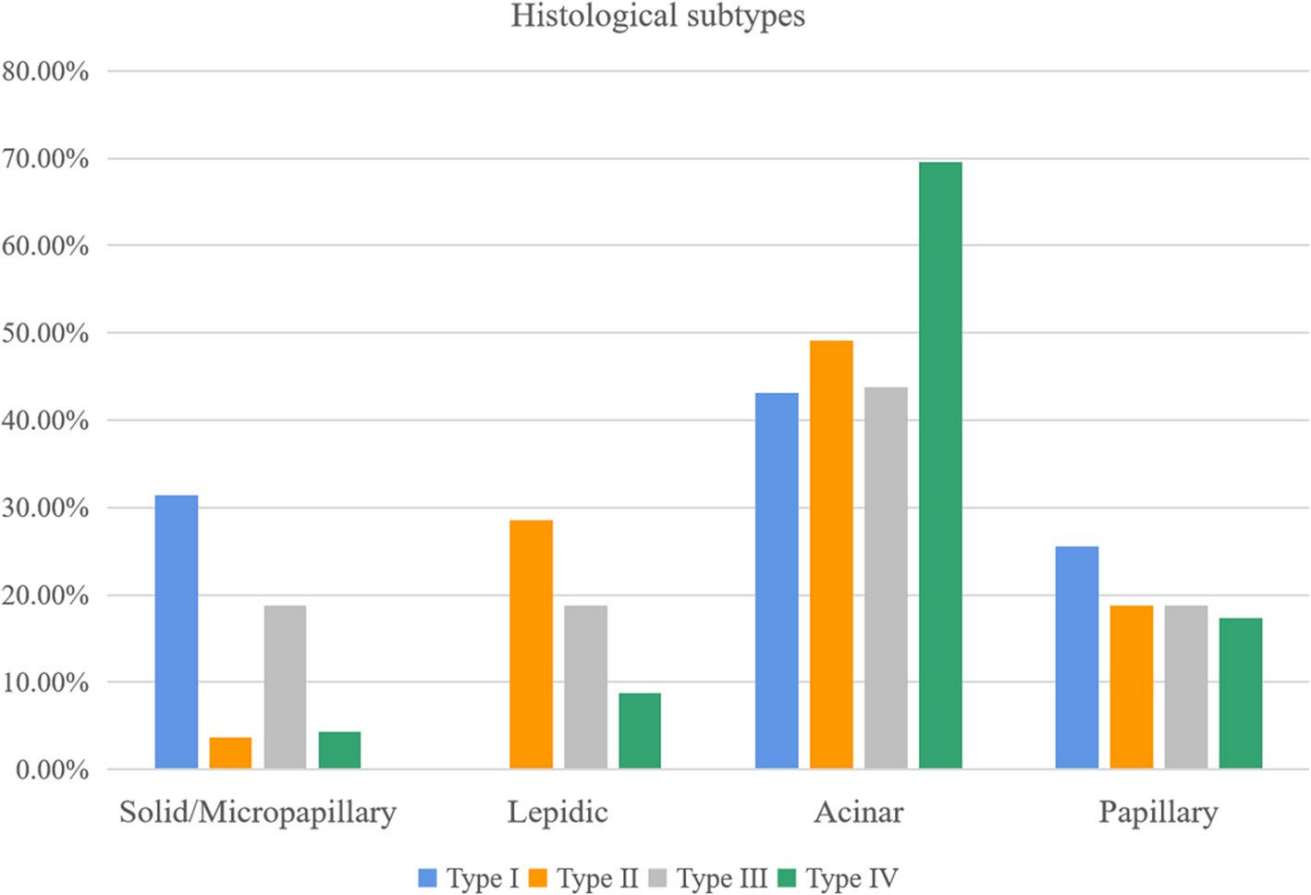
Distribution histogram of type I–IV SLADCs in different histological subtypes

Of all patients, histological classification was available for 202 patients who underwent surgical resection. As shown in Figs. 3 and 4 and Table 3, type I tumors had a greater proportion of the solid/micropapillary-predominant pattern as compared to type II, whereas the lepidic-predominant pattern was more dominant in types II and III than in type I (*p* < 0.05 each). However, there were no significant differences in the proportion of acinar-predominant and papillary-predominant patterns among all types (*p* > 0.05 each).

**Table 3:** Correlation between histological subtypes and morphological classification of SLADC

	Type I (*n* = 51)	Type II (*n* = 112)	Type III (*n* = 16)	Type IV (*n* = 23)	*p* value	Sig
Solid/micropapillary	16 (31.4%)	4 (3.6%)	3 (18.8%)	1 (4.3%)	< 0.001	I vs II
Lepidic	0 (0%)	32 (28.6%)	3 (18.8%)	2 (8.7%)	< 0.001	I vs II, I vs III
Acinar	22 (43.1%)	55 (49.1%)	7 (43.8%)	16 (69.6%)	0.191	-
Papillary	13 (25.5%)	21 (18.8%)	3 (18.8%)	4 (17.4%)	0.763	-

*SLADC* solid lung adenocarcinoma

### Correlation between gene mutation status and morphological classification of SLADC

The genetic mutation tests of EGFR, ALK, and KRAS were available for 36, 28, and 28 patients, respectively. Among them, 17 patients (*n* = 17/36; 47.2%) were positive for EGFR mutation, 1 patient (*n* = 1/28; 3.6%) tested positive for ALK mutation, and 7 patients (*n* = 7/28; 25%) tested positive for KRAS mutation. As shown in Table 4, types II and IV SLADCs were found to be correlated to EGFR mutation status, i.e., EGFR mutation rates were significantly higher in patients with types II and IV SLADCs than in those without (*p* < 0.05 each). However, no significant differences were observed in terms of ALK or KRAS mutation rates among the four tumor subtypes (*p* > 0.05 each).

**Table 4:** Correlation between gene mutation status and morphological classification of SLADC

Classification	Total	*EGFR* Positive	*EGFR* Negative	*p* value
Type I				0.083
Yes	9	2 (22.2%)	7 (77.8%)	
No	27	15 (55.6%)	12 (44.4%)	
Type II				0.036
Yes	13	9 (69.2%)	4 (30.8%)	
No	23	8 (34.8%)	15 (65.2%)	
Type III				0.065
Yes	10	2 (20.0%)	8 (80.0%)	
No	26	15 (57.7%)	11 (42.3%)	
Type IV				0.040
Yes	4	4 (100%)	0 (0%)	
No	32	13 (40.6%)	19 (59.4%)	
Classification	Total	*ALK* positive	*ALK* negative	*p* value
Type I				0.250
Yes	7	1 (14.0%)	6 (86.0%)	
No	21	0 (0%)	21 (100%)	
Type II				1.000
Yes	10	0 (0%)	10 (100%)	
No	18	1 (6.0%)	17 (94.0%)	
Type III				1.000
Yes	4	0 (0%)	4 (100%)	
No	24	1 (4.0)	23 (96.0)	
Type IV				1.000
Yes	7	0 (0%)	7 (100%)	
No	21	1 (5.0%)	20 (95.0%)	
Classification	Total	*KRAS* positive	*KRAS* negative	*p* value
Type I				0.141
Yes	7	0 (0%)	7 (100%)	
No	21	7 (33.0%)	14 (67.0%)	
Type II				0.674
Yes	10	3 (30.0%)	7 (70.0%)	
No	18	4 (22.0%)	14 (78.0%)	
Type III				0.253
Yes	4	2 (50.0%)	2 (50.0%)	
No	24	5 (21.0%)	19 (79.0%)	
Type IV				1.000
Yes	7	2 (29.0%)	5 (71.0%)	
No	21	5 (24.0%)	16 (76.0%)	

*SLADC* solid lung adenocarcinoma, *EGFR* epidermal growth factor receptor, *ALK* anaplastic lymphoma kinase, *KRAS* Kirsten rat sarcoma viral oncogene

### Survival analysis of patients with SLADC

**Fig. 5:**
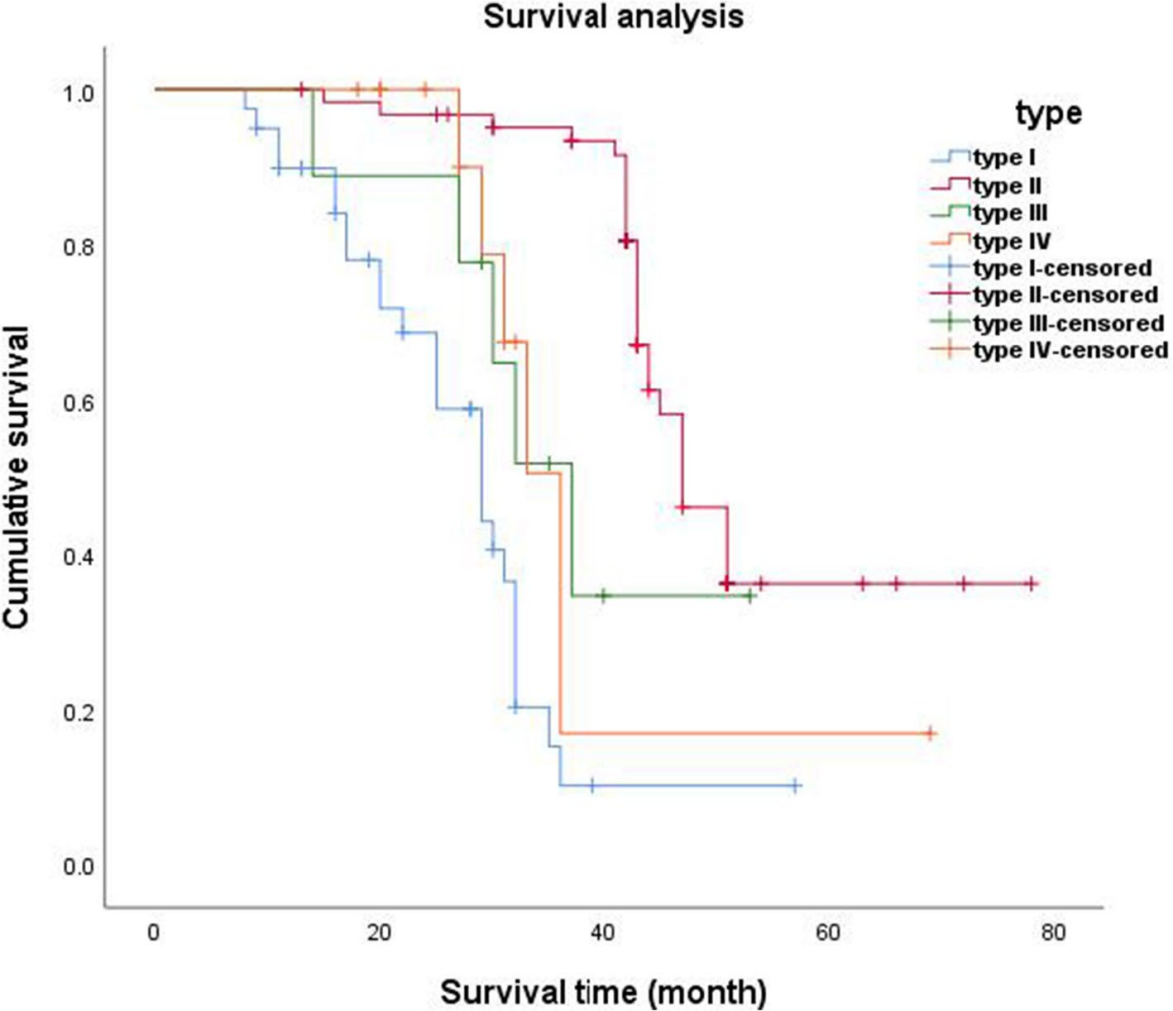
Survival analysis of patients with type I–IV SLADCs

Follow-up information was available for 126 of the SLADC patients undergoing surgical resection; among them, 29 patients (23%) experienced disease recurrence, and 14 patients (11.1%) died. Overall, the median DFS was 33 months; individually, the median DFS time for each tumor subtype was as follows: type I = 29 months, type II = 47 months, type III = 37 months, and type IV = 36 months. We found that the CT morphological classification was correlated with the DFS of patients. The Kaplan–Meier curves displayed those patients with type II SLADC had the longest DFS, followed by patients with types III and IV, and then patients with type I (i.e., II > III/IV > I) (*p* < 0.001 each) (Fig. 5). Among the 9 patients with type III tumors whose follow-up information was complete for survival analysis, their DFS correlated negatively with the thickness of cystic walls before operation (*r* =  − 0.69, *p* < 0.05).

## Discussion

This study offers a comprehensive understanding of the natural progression of SLADC, and the correlation between the CT-based morphological classification and pathological-molecular characteristics and surgical prognosis. We found that different SLADC morphological types present varying dynamic changes in follow-up CT scans, which has been a recent topic of discussion in this field. Chu et al. [5] analyzed the CT features of solid lung cancerous nodules with different sizes and stated that the tumors showed a more regular shape, lobulation, spiculation, vascular convergence, pleural retraction, and bronchial truncation as the nodule diameter increased. Saito et al. [6] evaluated the CT manifestations of subsolid nodules and found that GGO lesions tended to increase in size over time, with solid parts appearing in some lesions during follow-up examinations. Likewise, Tan et al. [20] investigated the CT characteristics and pathologic basis of solitary cystic lung cancer and found increased cystic airspace size, thickened cystic walls, and appearance of wall nodules at follow-up in 7 patients; in 2 patients, the cystic airspaces eventually became solid masses. Li et al. [21] analyzed the morphological evolution of 14 patients with localized pneumonic-type LADC and found that the tumor diameter enlarged over time, with increased attenuation and gradual disappearance of the air bronchogram, air space, and GGO component. All these reports concur with our results. Notably, unlike other types of SLADC, type II tumors observed in our study may have decreased in size during their progression which was usually accompanied by increased density or solid components; such changes have been reported in a few previous studies [22, 23]. Kaneda et al. [22] indicated that this phenomenon in type II tumors may be attributed to the alveolar collapse inside GGNs which may be transiently reversible and occurs before the appearance of fibrotic changes and invasion.

The VDT of pulmonary nodules measured by CT can be used to quantify their growth rates [9, 24–26], and familiarity with the morphological evolution and growth rates of these subtypes is crucial for the early diagnosis and rational development of treatment strategies. Some investigators have compared the VDTs for solid and subsolid malignant nodules [6, 24, 27]. Saito et al. [6] reported that the VDT of GGNs was significantly longer than that of solid nodules. Similarly, Aoki et al. [27] found that GGNs grew slowly, whereas solid nodules grew rapidly. Specifically, Park et al. [24] described the VDTs of solid and subsolid nodules as 248 and 657 days, respectively. In our study, the VDT of type I tumors was significantly shorter than that of type II, which is in line with the existing literature. However, to our knowledge, the VDT of types III and IV SLADCs has not been reported yet. The current study found that type I had the shortest VDT, followed by types IV and III, and then type II.

We also found that the histological subtypes varied among different SLADC types. The solid/micropapillary-predominant pattern was more frequent in type I tumors than in type II, while the lepidic-predominant pattern was more frequent in type II or III tumors than in type I. A few studies have reported that subsolid LADCs are strongly associated with a lepidic-predominant pattern, while solid LADCs are closely related to a non-lepidic-predominant pattern, which is consistent with our findings [28, 29]. It has also been reported that LADCs with different pathologic growth patterns have different growth rates [9, 24]. Specifically, a LADC with a solid/micropapillary-predominant pattern grows fastest, followed by that with an acinar/papillary-predominant pattern, and then that with a lepidic-predominant pattern. These findings also explain the VDT differences among different SLADC types observed in our study.

Additionally, the study revealed that type II and IV SLADC were correlated with EGFR-positive mutation. It is known that subsolid tumors have a high incidence of EGFR mutations, whereas solid tumors had a low incidence of EGFR mutations [30]. A recent study by Li et al. [4] showed that localized pneumonic-type LADC had a high EGFR mutation rate (76.6%). Accordingly, patients with advanced type II or IV SLADC are likelier to benefit from targeted therapy.

In addition to genetic mutations, we found that the CT-based morphological classification was related to the DFS of patients, i.e., patients with type II tumors had the longest DFS, followed by those with types III and IV tumors, and then those with type I tumors. It has been established that LADCs with a lepidic-predominant pattern tend to have a good prognosis and those with an acinar/papillary-predominant pattern usually have an intermediate prognosis, whereas a micropapillary/solid-predominant pattern is usually associated with a poor prognosis [31]. According to our findings, type I tumors had a high proportion of the micropapillary/solid-predominant pattern, whereas the lepidic-predominant pattern was more common in types II and III, which corroborates the prognoses reported for each SLADC type. Furthermore, we found that a thick cystic wall was indicative of a poor prognosis in patients with type III tumors.

Our study had certain limitations. First, our analyses were limited to SLADC, and other histological types of lung cancer were not included. Second, the retrospective nature and strict inclusion–exclusion criteria used in this study might have led to selection bias. Moreover, it was difficult to maintain homogenous intervals of CT follow-ups among different types of SLADC. Third, given the small number of patients who received genetic mutation tests here, future studies with a larger sample size are needed to substantiate our findings.

In conclusion, our findings demonstrate that SLADCs with different morphological presentations exhibit varying natural progression, histological subtypes, gene mutation status, and surgical prognosis. A good understanding of these features can help make accurate diagnoses, develop individual treatment strategies, and predict patient outcomes.

### Supplementary Information

**Additional file 1: Supplementary Table 1.** ICC values for CT features of type I-IV tumors between two observers.

## Data Availability

The datasets used or analyzed during the current study are available from the corresponding author on reasonable request.

## Abbreviations

ALK: Anaplastic lymphoma kinase
CT: Computed tomography
DFS: Disease-free survival
EGFR: Epidermal growth factor receptor
GGN: Ground-glass nodule
GGO: Ground-glass opacity
ICC: Intraclass correlation coefficient
KRAS: Kirsten rat sarcoma viral oncogene
PACS: Picture archiving and communication
ROI: Region of interest
SLADC: Solitary lung adenocarcinoma
VDT: Volume doubling time
VOI: Volume of interest

## References

[1] Siegel RL, Fedewa SA, Miller KD (2015). Cancer statistics for Hispanics/Latinos, 2015. CA Cancer J Clin.

[2] Jeudy J, White CS, Munden RF (2008). Management of small (3–5mm) pulmonary nodules at chest CT: global survey of thoracic radiologists. Radiology.

[3] Travis WD, Brambilla E, Noguchi M (2011). International association for the study of lung cancer/American Thoracic Society/European Respiratory Society international multidisciplinary classifification of lung adenocarcinoma. J Thoracic Onco.

[4] Li Q, He XQ, Fan X, Luo TY, Huo JW, Huang XT (2021). Computed tomography morphological classification of lung adenocarcinoma and its correlation with epidermal growth factor receptor mutation status: a report of 1075 cases. Int J Gen Med.

[5] Chu ZG, Zhang Y, Li WJ, Li Q, Zheng YN, Lv FJ (2019). Primary solid lung cancerous nodules with different sizes: computed tomography features and their variations. BMC Cancer.

[6] Saito H, Yamada K, Hamanaka N (2009). Initial findings and progression of lung adenocarcinoma on serial computed tomography scans. J Comput Assist Tomogr.

[7] Hasegawa M, Sone S, Takashima S (2000). Growth rate of small lung cancers detected on mass CT screening. Br J Radiol.

[8] Godoy MC, Sabloff B, Naidich DP (2012). Subsolid pulmonary nodules: imaging evaluation and strategic management. Curr Opin Pulm Med.

[9] Hong JH, Park S, Kim H (2021). Volume and mass doubling time of lung adenocarcinoma according to WHO histologic classification. Korean J Radiol.

[10] Travis WD, Brambilla E, Noguchi M (2011). International association for the study of lung cancer/american thoracic society/european respiratory society international multidisciplinary classification of lung adenocarcinoma. J Thorac Oncol.

[11] Sakurai H, Asamura H, Miyaoka E (2014). Differences in the prognosis of resected lung adenocarcinoma according to the histological subtype: a retrospective analysis of Japanese lung cancer registry data. Eur J Cardiothorac Surg.

[12] Inamura K (2018). Clinicopathological characteristics and mutations driving development of early lung adenocarcinoma: tumor initiation and progression. Int J Mol Sci.

[13] Saito M, Suzuki H, Kono K, Takenoshita S, Kohno T (2018). Treatment of lung adenocarcinoma by molecular-targeted therapy and immunotherapy. Surg Today.

[14] Ahmed SM, Salgia R (2006). Epidermal growth factor receptor mutations and susceptibility to targeted therapy in lung cancer. Respirology.

[15] Miyahara N, Nii K, Benazzo A (2019). Solid predominant subtype in lung adenocarcinoma is related to poor prognosis after surgical resection: a systematic review and meta-analysis. Eur J Surg Oncol.

[16] Urer HN, Kocaturk CI, Gunluoglu MZ (2014). Relationship between lung adenocarcinoma histological subtype and patient prognosis. Ann Thorac Cardiovasc Surg.

[17] Ye T, Deng L, Xiang J (2018). Predictors of pathologic tumor invasion and prognosis for ground glass opacity featured lung adenocarcinoma. Ann Thorac Surg.

[18] Mehrara E, Forssell-Aronsson E, Ahlman H, Bernhardt P (2007). Specific growth rate versus doubling time for quantitative characterization of tumor growth rate. Cancer Res.

[19] Travis WD (2014). The 2015 WHO classification of lung tumors. Pathologe.

[20] Tan Y, Gao J, Wu C (2019). CT characteristics and pathologic basis of solitary cystic lung cancer. Radiology.

[21] Li Q, Fan X, Huo JW, Luo TY, Huang XT, Gong JW (2022). Differential diagnosis of localized pneumonic-type lung adenocarcinoma and pulmonary inflammatory lesion. Insights Imaging.

[22] Kaneda H, Nakano T, Taniguchi Y, Saito T, Konobu T, Saito Y (2014). A decrease in the size of ground glass nodules may indicate the optimal timing for curative surgery. Lung Cancer.

[23] Lee JH, Park CM, Lee SM, Kim H, McAdams HP, Goo JM (2016). Persistent pulmonary subsolid nodules with solid portions of 5 mm or smaller: their natural course and predictors of interval growth. Eur Radiol.

[24] Park S, Lee SM, Kim S (2020). Volume doubling times of lung adenocarcinomas: correlation with predominant histologic subtypes and prognosis. Radiology.

[25] Devaraj A, van Ginneken B, Nair A, Baldwin D (2017). Use of volumetry for lung nodule management: theory and practice. Radiology.

[26] Kanashiki M, Tomizawa T, Yamaguchi I (2012). Volume doubling time of lung cancers detected in a chest radiograph mass screening program: comparison with CT screening. Oncol Lett.

[27] Aoki T, Nakata H, Watanabe H (2000). Evolution of peripheral lung adenocarcinomas: CT findings correlated with histology and tumor doubling time. AJR Am J Roentgenol.

[28] Vazquez M, Carter D, Brambilla E (2009). Solitary and multiple resected adenocarcinomas after CT screening for lung cancer: histopathologic features and their prognostic implications. Lung Cancer.

[29] Lederlin M, Puderbach M, Muley T (2013). Correlation of radio- and histomorphological pattern of pulmonary adenocarcinoma. Eur Respir J.

[30] Huo JW, Luo TY, He XQ, Gong JW, Lv FJ, Li Q (2022). Radiological classification, gene-mutation status, and surgical prognosis of synchronous multiple primary lung cancer. Eur Radiol.

[31] Yoshizawa A, Motoi N, Riely GJ (2011). Impact of proposed IASLC/ATS/ERS classification of lung adenocarcinoma: prognostic subgroups and implications for further revision of staging based on analysis of 514 stage I cases. Mod Pathol.

