# Supplementary material for: Solitary lung adenocarcinoma: follow-up CT, pathological-molecular characteristics, and surgical prognosis for different morphological classifications
Source: Insights Imaging. 2023 Nov 27;14:209. doi: 10.1186/s13244-023-01563-x (PMC10682316; doi:10.1186/s13244-023-01563-x)
Supplement: Supplementary file 1 — Additional file 1: Supplementary Table 1. ICC values for CT features of type I-IV tumors between two observers. [file 13244_2023_1563_MOESM1_ESM.docx]

**Solitary lung adenocarcinoma: follow-up CT, pathological-molecular characteristics, and surgical prognosis for different morphological classifications**

**ELECTRONIC SUPPLEMENTARY MATERIAL**

The specific definition of CT features analyzed and measured by radiologists were as follows:

(1) location (left upper and lower lobes or right upper, middle and lower lobes); (2) size (longest diameter of tumor in the lung window setting); (3) shape (regular [round or oval] or irregular [having an uneven contour that cannot be classified as round or oval]); (4) boundary (well-defined or ill-defined); (5) lobulation (irregular margins with arc-shaped swelling); (6) s[piculatio](javascript:;)n (small burrs protruding around the lesion); (7) CT value of tumor (largest section of the tumor for each patient was selected from axial CT images in the lung window setting [window width: 1600HU; window level: −600HU]; for tumor with homogeneous density, a circular region of interest [ROI] was drawn to include more than half of the entire tumor, while for tumors with heterogeneous density, two circular ROIs as large as possible were placed on ground-glass opacity [GGO] and solid regions of the lesion, cautiously avoiding air space and vessels within the tumor); (8) air bronchogram (branched or tubular air structure within tumors); (9) air space (round or oval air attenuation within the tumor); (10) pleural retraction (linear structures connected between the tumor and pleura); (11) pleural attachment (tumor attached to the pleura with the margin obscured by the pleura); (12) vessel convergence sign (whether one or more vascular structures were pulled toward or through the tumors); (13) lymphadenopathy (hilar or mediastinal lymph nodes with short-axis diameter of >1 cm); (14) pleural effusion (fluid accumulation in the pleural cavity); (15) cyst wall thickness (measured from the thickest part of the cyst wall); (16) septation within the cyst (linear separation within the cystic space); (17) GGO component (GGO mixed with consolidation or well-defined GGO around consolidation).

**Supplementary Table 1.**ICC values for CT features of type I-IV tumors between two observers

| Type I | ICC values | Type II | ICC  values | Type III | ICC  values | Type IV | ICC  values |
| --- | --- | --- | --- | --- | --- | --- | --- |
| Location  Size  Shape  Lobulation  [Spiculation](javascript:;)  Air bronchogram sign  Air space  Pleural retraction  Pleural attachment  Vessel convergence sign  Lymphadenopathy  Pleural effusion | 1.000  0.954  0.962  0.936  0.930  0.978  0.969  0.930  0.978  0.898  0.920  1.000 | Location  Size  Shape  CT value of tumor  Lobulation  [Spiculation](javascript:;)  Air bronchogram sign  Air space  Pleural retraction  Pleural attachment  Vessel convergence sign  Lymphadenopathy  Pleural effusion | 1.000  0.958  0.956  0.936  0.945  0.945  0.965  0.977  0.939  0.986  0.899  0.945  1.000 | Location  Size  Shape  Cyst walls thickness  Septation within the cyst  GGO component  Pleural retraction  Pleural attachment  Vessel convergence sign  Lymphadenopathy  Pleural effusion | 1.000  0.901  1.000  1.000  1.000  1.000  1.000  1.000  0.828  1.000  1.000 | Location  Size  Shape  CT value of tumor  Air bronchogram sign  Air space  GGO component  Pleural retraction  Pleural attachment  Vessel convergence sign  Lymphadenopathy  Pleural effusion | 1.000  0.941  1.000  0.939  1.000  1.000  1.000  1.000  1.000  0.904  1.000  1.000 |

*ICC* intraclass correlation coefficient, *CT* computed tomography, *GGO* ground-glass opacity
